# Quantitative and Real‐Time Evaluation of Human Respiration Signals with a Shape‐Conformal Wireless Sensing System

**DOI:** 10.1002/advs.202203460

**Published:** 2022-09-11

**Authors:** Sicheng Chen, Guocheng Qian, Bernard Ghanem, Yongqing Wang, Zhou Shu, Xuefeng Zhao, Lei Yang, Xinqin Liao, Yuanjin Zheng

**Affiliations:** ^1^ School of Electrical and Electronic Engineering Nanyang Technological University 50 Nanyang Avenue Singapore 639798 Singapore; ^2^ Visual Computing Center King Abdullah University of Science and Technology Thuwal 23955‐6900 Kingdom of Saudi Arabia; ^3^ School of Geophysics and Information Technology China University of Geosciences Beijing 100084 P. R. China; ^4^ Shanghai Institute of Intelligent Electronics & Systems School of Microelectronics Fudan University Shanghai 200433 P. R. China; ^5^ Key Laboratory of Education Ministry for Modern Design and Rotor‐Bearing System Xi'an Jiaotong University Xi'an 710049 P. R. China; ^6^ School of Electronic Science and Engineering Xiamen University 422 Siming South Road Xiamen 361005 P. R. China

**Keywords:** machine learning, physiological status monitoring, respiration signal, wireless sensing system

## Abstract

Respiration signals reflect many underlying health conditions, including cardiopulmonary functions, autonomic disorders and respiratory distress, therefore continuous measurement of respiration is needed in various cases. Unfortunately, there is still a lack of effective portable electronic devices that meet the demands for medical and daily respiration monitoring. This work showcases a soft, wireless, and non‐invasive device for quantitative and real‐time evaluation of human respiration. This device simultaneously captures respiration and temperature signatures using customized capacitive and resistive sensors, encapsulated by a breathable layer, and does not limit the user's daily life. Further a machine learning‐based respiration classification algorithm with a set of carefully studied features as inputs is proposed and it is deployed into mobile clients. The body status of users, such as being quiet, active and coughing, can be accurately recognized by the algorithm and displayed on clients. Moreover, multiple devices can be linked to a server network to monitor a group of users and provide each user with the statistical duration of physiological activities, coughing alerts, and body health advice. With these devices, individual and group respiratory health status can be quantitatively collected, analyzed, and stored for daily physiological signal detections as well as medical assistance.

## Graphical abstract

This work develops a soft, wireless, and non‐invasive device for quantitative and real‐time evaluation of human respiration process. By further proposing a machine learning‐based respiration classification algorithm to accurately recognize body status, individual and group respiratory status can be quantitatively collected, analyzed and stored on system deployed clients for daily physiological signal detections as well as medical assistance.

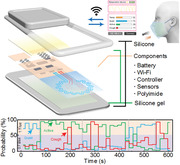

## 1 Introduction

Respiration signals are vital signs of life, especially when taking the sweeping changes caused by several respiratory‐infectious diseases worldwide into consideration.^[^ ^1^, ^2^, ^3^ ^]^ Severe acute respiratory syndrome coronavirus, influenza virus subtype H5N1 and coronavirus disease 2019 are all respiratory‐related diseases, that can induce pneumonia‐like symptoms ranging from a severe inability to breathe to mild coughing.^[^ ^4^, ^5^, ^6^ ^]^ The infected patients should be monitored by measuring their respiration frequently,^[^ ^7^ ^]^ and quickly be transferred to designated higher‐level hospitals if respiration rates reach 30 breaths per min or higher.^[^ ^8^ ^]^ Patients may still suffer from cough after contracting and recovering from the respiratory disease. The dosage of treatments sometimes needs to be adjusted according to the degree of cough, which is currently assessed by visual observation. Daily respiration monitoring also reflects changes in sympathetic nervous system activity stemming from underlying health conditions. For example, the respiration is related to defects in the central nervous system,^[^ ^9^ ^]^ infarction of medulla,^[^ ^10^ ^]^ and cardiovascular functions.^[^ ^11^ ^]^ Therefore, continuous measurement of respiration signals with portable devices is a powerful route for non‐invasive health monitoring in many cases, but has not yet been fully exploited.

Several respiration‐sensing devices for the static monitoring of human respiration signals have been developed,^[^ ^12^, ^13^, ^14^, ^15^ ^]^ including triboelectric and resistive sensors for identifying respiration rates before and after physical exercise. Unfortunately, existing devices are far from portable, compact and smart enough, and do not meet the needs for efficient signal collection and processing in complex environments such as frequent moving in daily interactions,^[^ ^16^, ^17^, ^18^ ^]^ without restricting the user's freedom.^[^ ^19^ ^]^ Besides, software associated with the device that is able to identify a physiological status especially an abnormal status such as intensive coughing is yet to be developed.

There are, in particular, three key challenges to address. First, such non‐invasive measures are prone to both sensitivity and anchoring bias, thus the signal quality is often affected by the temperature parameters and unsatisfactory.^[^ ^13^ ^]^ Second, despite the availability of several wireless readout wearable devices,^[^ ^20^, ^21^, ^22^, ^23^ ^]^ it is still hard to maintain reliable data acquisition quality under shape deformation owing to the increase of electrical resistance between electronic components. It is thus difficult to build a completely wearable system that is capable of processing multi‐channel inputs using miniaturized portable readout circuits. Third, achieving a high identification accuracy of abnormal physiological status from complex respiration behaviors, for example, the interval coughing from continual speaking, is definitely not an easy task. The widely diverse characteristics of individual respiration habits also represents additional unsolved challenges.^[^ ^24^ ^]^ In general, current devices and strategies do not offer sufficient performance or practicality for routine use in respiration diagnosis.

Here, we report a strategy that exploits a soft, wireless, and low‐profile device capable of collecting both respiration and temperature signatures of physiological processes at a commercially viable data quality in a non‐invasive way. This device softly couples to a wearing mask or a face shield to enable the capture of signals with self‐error‐correcting functions, providing a more comprehensive perspective on human body conditions. Electron beam (e‐beam) evaporation technology is applied for rapid and mass production of sensor patterns to reduce personal training and process optimization.^[^ ^25^, ^26^, ^27^ ^]^ Breathable films are used to encapsulate both the resistive temperature sensor (R‐TS) and capacitive respiration sensor (C‐RS). Using device data collected under well‐defined conditions (sitting, sleeping, coughing, exercising, and speaking) and an associated understanding of the respiration physics, we derived a feature defined as respiration stability, which proves to be more suitable for uniquely characterizing complex human physical activities. The training study involves systematic investigation and data analysis to develop a machine leaning (ML) algorithm to categorize and quantify respiration signals, followed by validation of various behavioral statuses with data analytics approaches. Furthermore, a confirmatory study validates the device performance and the algorithm in a cohort of volunteers (*n* = 13, including females and males from different nationalities). The proposed algorithm compares well to the manually labelled results defined by direct visual observation and camera recordings.

## 2 Results and Discussion

### 2.1 Engineering Mechanics of the Device

**Figure 1:**
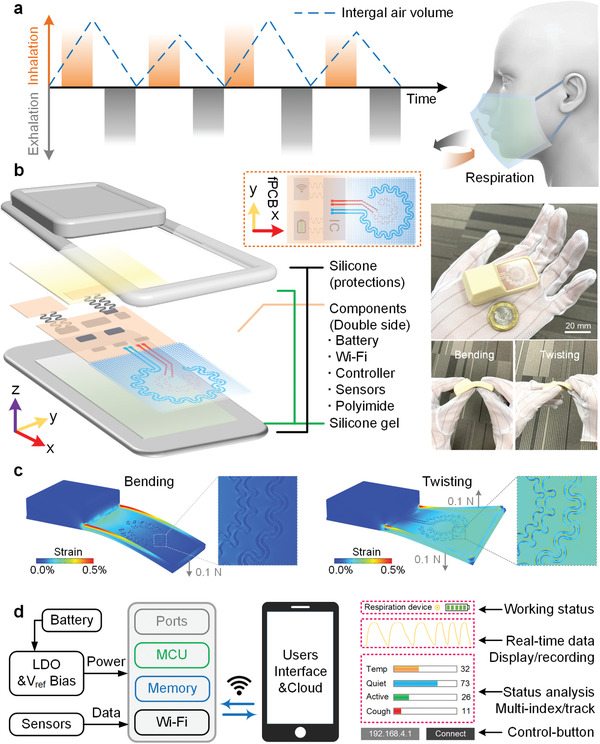
Main idea of the designed wireless sensing system. a) Schematic view of the cycled respiration process. b) Exploded schematic illustration of the active subsystems, enclosure architectures, and customized sensors. Images that demonstrate soft‐device mechanics with shape‐deformation. c) Finite element modelling of the mechanics during bending and twisting deformations. d) Block diagram of the system operation with user interface (Note S1, Supporting Information).

Physiological respiration involves cycled air exchanges of inhaled and exhaled breaths, which are defined as a positive column (orange) and negative column (grey) in **Figure** **1**a, respectively. Compared with the indirect post‐processing respiration signals using skin electrodes,^[^ ^28^ ^]^ direct sensing of the integral air volume provides more intuitive information, high data fidelity and non‐irritating interfaces. Figure 1b outlines the overall device layout, with images that demonstrate its ability to adapt naturally with attached surface deformation. The design incorporates paired customized sensors (a 0.1 µm thick R‐TS and a 1 µm thick C‐RS as shown in the orange dashed box), a 1 µm thick breathable encapsulation, peripheral electronic components and a hollow silicone pocket with strain‐isolated layers. The electronic part consists, more specifically, of a flexible printed circuit board (fPCB) based on an 80‐µm thick middle polyimide supporting layer with 18 µm thick patterned copper (Cu) traces on the top and bottom surfaces. Immersion gold is used upon annealed Cu for more reliable solderability in the extra process. The electronic subsystems mainly include: i) a customized readout circuit for simultaneously resistance and capacitance sensing, with strong anti‐interference ability; ii) a microcontroller (ATmega 328P) for acquiring data from the reading circuit and communicating the results wirelessly via low‐powered WiFi protocols; and iii) a rechargeable 150 mAh lithium‐ion polymer battery with programmed wireless charging function. As these subsystems highly rely on rigid, planar off‐the‐shelf components, they should be subtly integrated in a manner that simultaneously offers soft, shape‐compatible mechanics as well as effective coupling to the body respiration. The schemes used here exploit advanced versions of design concepts in stretchable electronics,^[^ ^21^, ^29^ ^]^ adapted for use with the fPCB generally, and for the interconnects between the subsystems specifically (Figure S1, Supporting Information). Pre‐buckled interconnects mechanically and electrically joint three rectangular islands (battery island of 25 × 23 mm^2^, fPCB island of 25 × 49 mm^2^ and sensors island of 25 × 25 mm^2^) as shown in Figure 1b. Small pieces of flexible cured silicone‐gel sheets (smooth‐on, 50 µm thick, 0.1 MPa Young's modulus) are used to ensure the insulation features between neighboring islands.

The R‐TS and C‐RS rested under the sunroof of a silicone pocket (50% of the overall area of the system) to support efficient respiration recordings with minimal mechanical constraints from other parts of the system (Figure 1c). Step‐by‐step e‐beam evaporation technology (layered Cu, 110 GPa) is applied for potential high‐volume manufacturing (Figure S2, Supporting Information). Nevertheless, to prevent possible deformation failure and achieve superior sensing abilities, both sensor structures are designed to be serpentine‐shaped patterns on a 50 µm thick polyimide film (Figure S3, Supporting Information). Finite element analysis highlights considerable mechanical advantages of these arc‐shaped geometries to assume traction‐free architectures that absorb tensile deformations in the illustrated modes. In the optimized design structure, the maximum effective strain in the Cu layer is significantly less than the yield strain (0.3%) under various mechanical loads (bending and twisting without any supporting stiffness). Moreover, the circular distribution of these serpentine structures allows for a more sensitive capture of respiration signals within certain areas to the less‐serpentine design (Figures S4 and S5, Supporting Information). A reference voltage module (Vref, 8 ppm/°C with ±0.05% accuracy) is combined with the low‐dropout regulator (LDO) for precise resistive measurements (Figure 1d). The layout for these components can be seen in Figure S6, Supporting Information. All the tested data would be processed and presented on a compact user interface for revealing real‐time individual physiological parameters and possible health‐condition alarming. These results highlight the customized sensors, subtly integrated circuits, and reasonable designed layouts necessary to accommodate realistic user requirements with a stable and reliable sensing ability.

### 2.2 Data Collection with Customized Sensors

**Figure 2:**
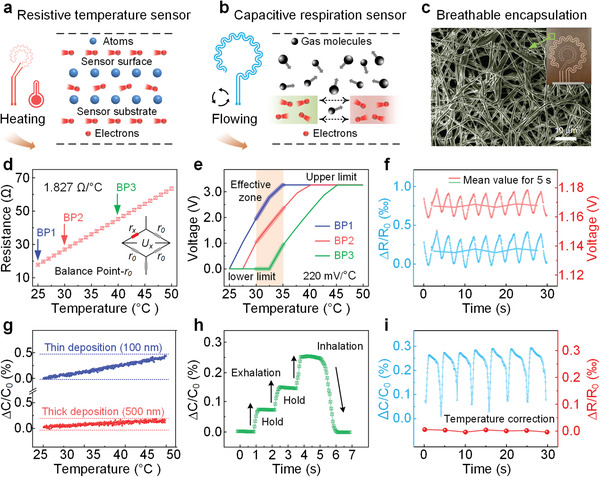
Design and characterization of the customized sensors. a) Mechanisms of temperature sensing and b) respiration sensing. c) SEM image of an encapsulation layer with an inset photographic image. d) Calibration plot of the R‐TS in the physiological temperature range (*n* = 5 trials per design, error bars = ±*σ*). e) Output voltage of readout circuits with certain BPs. f) Real‐time respiration measurement with R‐TS. g) Basic capacitance of C‐RS influenced by temperature. h) Dynamic response of C‐RS with controlled conditions. i) Real‐time respiration measurement with C‐RS.

E‐beam evaporation technology enables rapid metal coating on electronics with low cost and mass production. For the deposited Cu layer in our R‐TS, the conductivity decreases along with higher temperature owing to the greater collision chance between free electrons and surrounding atoms (**Figure** **2**a). Although such mechanisms can reveal the temperature variation, these metal‐based sensors usually have quite a small basic resistance and thereby slight temperature‐induced fluctuations. We explore two effective means to address this dilemma. One is to develop more sensitive peripheral readout circuits, where multi‐stage amplifiers are used combing with Wheatstone bridge connections (Figure S7, Supporting Information). Note that, the matching resistor decides the balance point (BP) of the bridge, which would be introduced in Figure 2e for proper selection. The other one is to reduce the film thickness (as low as 100 nm) for obtaining a higher basic resistive value. As for the C‐RS, the inner distributed electrical field is regulated by the passing gas molecules (Figure 2b), which is quantitatively explained in Note S2, Supporting Information, and our recent work.^[^ ^25^ ^]^ Both resistive and capacitive sensors have their own advantages to achieve the desired sensor performance: a resistive sensor can yield high temperature response for the system calibration and a capacitive sensor is less susceptible to temperature variations. Therefore, a complementarity would benefit the quality and fidelity of the signals. To ensure the safety of the system while improving the breathability for a more sensitive perception, a 1 µm thick breathable and transparent layer prepared by electron‐spinning technology is covered upon the sensor pair (Figure 2c), which is also marked in the inset image. The fiber‐stacked structure can be observed using a field emission scanning electron microscope (FESEM) and contributes to the efficient transportation of respiration air. The R‐TS shows a fast, accurate, and stable response to temperature variations with a sensitivity of 1.827 Ω/°C within a wide detection range (Figure 2d). To determine the proper BP of the Wheatstone bridge, the basic resistive values under 25, 30, and 40 °C are taken into consideration (BP1, BP2, and BP3, respectively). Figure 2e describes the multi‐amplified output voltage at given BPs. In the effective detection zone (from 30 to 35 °C, slightly lower than human body temperature), BP2 shows better performance than BP1 and BP3, while BP1 and BP3 reach the upper and lower limitations within this range, respectively. Note that, too small or too large amplification factors might impair the sensitivity or narrow the detection range with electrical signals that are difficult to eliminate. Regarding the output voltage sensitivity of 220 mV/°C here, the maximum detection range is 15 (3.3 V/220 mV)°C. The real‐time monitoring temperature trace, relative resistance change, (∆*R*/*R*_0_, and ∆*R* = *R* − *R*_0_, where *R*_0_ represents the initial sensor resistance and *R* is the temperature‐dependent resistance) is shown in Figure 2f, where a periodical wave can be seen with corresponding output voltage. For better user experience, the data only outputs a statistical mean value every 5 s. Figure 2g indicates the basic relative capacitance (∆*C*/*C*_0_, and ∆*C* = *C* − *C*_0_, where *C*_0_ represents the initial sensor capacitance and *C* is the respiration‐dependent capacitance) of 100 and 500 nm thick C‐RS from 25 to 50 °C. We can see that the thick deposited sensor is less influenced by external temperature fluctuation, benefited from a reduced real part of sensor impedance. That makes an opposite deposition request to the R‐TS, therefore step‐by‐step deposition technology is explored to meet individual needs here. The mechanisms for sensitive capacitive‐reading circuits with high resolution are shown in Figure S8, Supporting Information. Using the 100 nm thick C‐RS, dynamic respiration signals consistent with controlled volunteer behavior can be collected (Figure 2h). The capacitance increases after each exhalation, stays still during the temporary breath‐holding, and goes back after the inhalation. Cycled respiration signals are illustrated in Figure 2i (after temperature correction, described in Note S3, Supporting Information), indicating more details and higher accuracy compared with the data in Figure 2f. Considering the unique properties of R‐TS and C‐RS, they are carefully fabricated in pair for ideally suitable monitoring of the human respiration and following data processing.

### 2.3 Data and Analysis Approaches for Measurement from the Device

**Figure 3:**
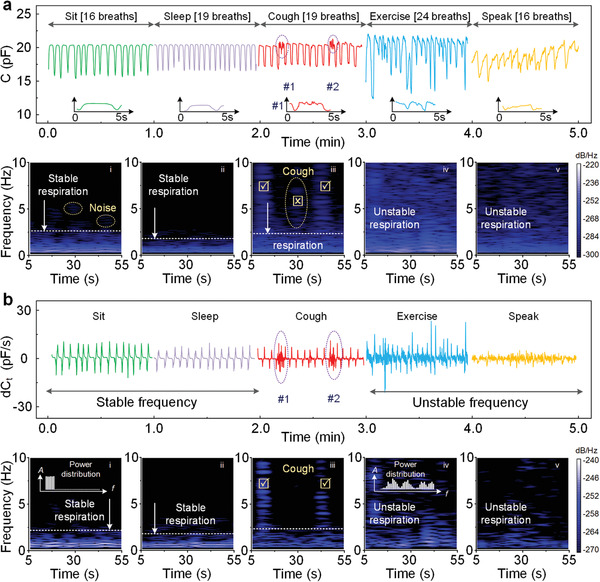
Understanding of the signal features and proposing an effective pre‐processing method. a) Comparison of respiration signals captured by the sensing system during five controlled conditions as time series and spectrogram plots. Partially enlarged details are also placed under the corresponding curve. The color indicates the amplitude spectral density. The frequency analysis uses a Hanning window with a frame size of 12.8 s moving in time step of 0.05 s. b) Data processed from same raw data and corresponding plots in time domain. The defined respiration stability is an effective physiological parameter for data processing.

Natural physiological processes generate diverse respiration signals, with the normal respiration rates of less than 30 breaths per minute (<0.5 Hz) and other featured signs.^[^ ^30^ ^]^ **Figure** **3**a illustrates time series and spectrogram representations of the collected raw data under five controlled conditions (sitting, sleeping, coughing, exercising, and speaking) from one volunteer. Each event lasts for 60 s followed by a short interval. The spectrograms use a Hanning window with a frame size of 12.8 s and an overlapping duration of 12.7 s. These identifying features extend in frequency up to 10 Hz, but with the most notable amplitude in the range of 0–1 Hz (normal respiration rates). The sit signals feature high‐quality harmonic structures with fundamental rates of 16 breaths in the normal range of typical adults. Respiration activities, including exhalation and inhalation, give periodic pulses with rising and decreasing trends. The power concentrates in a frequency band of 0–1 Hz as seen in the corresponding spectrogram. Some small noise (>1 Hz) might be observed if the body moves accidently. Sleeping events also produce stable respiration signals with a bit 19 breaths per min under the regulation of the autonomic nervous system.^[^ ^31^ ^]^ While the respiration frequency is thought to be mostly correlated with heart rate or blood pressure,^[^ ^31^ ^]^ the simulating coughing signals demonstrate higher‐frequency fluctuations (#1 and #2) in the curve associated with aerosol droplets spraying from mouth and nose. Such fluctuations can be revealed by the power concentration change in the frequency domain (1–10 Hz, the check marks in Figure 3a‐iii). Note that, each cough duration should be more than 1 s as a valid tag. Besides the marked timings, we can see that a vague peak (the cross mark in Figure 3a‐iii) lies between the two coughs caused by a small interference, which might impair the training accuracy. For the respiration data of exercising and speaking, signal amplitudes show more undulations with higher power concentration in the rest of the stable respiration zone in spectrograms. The talking and sports activity would interfere with our breathing rhythm in most occasions. However, these signals should not be classified into the abnormal coughing samples, even though there are considerable power distributions between 1 and 10 Hz. A ML based method can be used to classify the aforementioned human body status from the raw respiration signal.^[^ ^32^, ^33^, ^34^ ^]^ However, such a naive method is proven not able to yield an accurate enough prediction through our experiments. A major reason for the inaccurate prediction is due to the shifting signal baselines and varying amplitudes across the subjects (gender, age, body type and user devices, see Figure S9, Supporting Information). To alleviate this issue, we propose to extract the relative capacitance gain as the input instead of the raw signal to the ML algorithm. We denote the relative capacitance gain as d*C_t_* = *δC_t_*/*C_t_* = (*C_t_*_+1_ − *C_t_*)/*C_t_*, where *C_t_*_+1_ and *C_t_* stand for the capacitance value collected on the timing of *t*+1 and *t*. After this pre‐processing, all signals have a constant baseline (equal to 0) and relatively close magnitudes for each activity category. The plotted data after pre‐processing and the corresponding time‐frequency diagrams are presented in Figure 3b. The quiet physiological status, including sitting and sleeping here, still show stable periodic pulses symmetrically distributed on both sides of the time axis. Power distributions are more condensed within the stable respiration zone. Coughing signatures clearly appear in the Figure 3b‐iii with less noise (above vague peak in Figure 3a‐iii). Active physiological status, including exercising and speaking here, also have the same signal baseline and generate erratic power distributions in the unstable respiration zone. In practice, respiration signals consist of data streams that superpose body information from a multitude of sources besides the demonstrated events in Figure 3. We mostly focus on the key human activities (quiet and active) and coughing status. With the pre‐processing step, a high recognition accuracy of over 90% is achieved. Figure S10, Supporting Information, also presents the device outputs before and after shape deformation conditions, where the baseline‐shift can be effectively weakened, benefitted by this derivation step.

### 2.4 An ML Algorithm for Automated Human Body Status Detection

**Figure 4:**
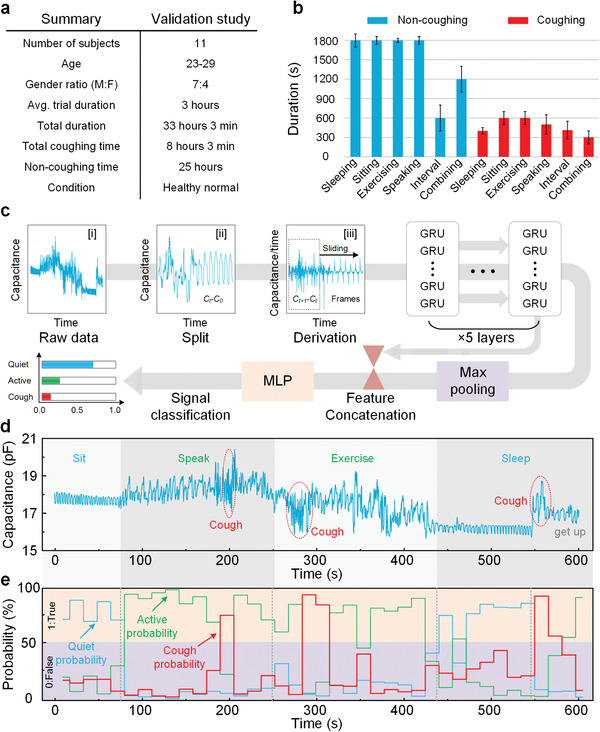
Overview of datasets, signal processing pipelines, and validation methods. a) The algorithm validation data was collected from 11 healthy normal volunteers. b) Activities performed by each participant and their corresponding activity durations. The physical activities involve sleeping, sitting, exercising, and speaking. The interval duration is also counted to obtain more precise sample splits. c) The proposed GRU‐based framework for respiration signal classification. Raw respiration signals are segmented into frames. The d*C* parameter is extracted as the feature set. The output index can indicate real‐time body status along with the sliding frame. d) Time series data of our validation cohort with a single subject performing various physical activities, including sitting, speaking, exercising, sleeping and waking up. This process is simulated by the participant in only 600 s. e) The output probability for a state is characterized as “True” if the probability exceeds 50%. The red, green and purple lines indicate the probability of coughing, quiet and active status for each frame of 30 s with 50% overlapping, respectively. The highest probability represents the dominating body status if all the values are less than 50%.

With the comprehensive understanding of respiration signal features, an ML algorithm is proposed for automated detection of human body status among being quiet, active and coughing. A respiration classification dataset contains respiration data from 5 types of activities (sleeping, sitting, exercising, speaking, and combinations with and without coughing during each condition) was collected from 11 subjects to train and validate the proposed ML algorithm (**Figure** **4**a,b). The annotated signals from a random subject were chosen as the validation set while the others were used in training. The ML model was trained on the training set and validated on the validation set, and the best performed model on the validation set was picked as the final model. The model is built upon the gated recurrent unit (GRU), as shown in Figure 4c. GRU is a widely‐used neural network module that is suitable for dealing with events with large interval and delay in time series, thus appropriately meeting the needs of cough‐like signals detection. Moreover, the GRU model possesses a fast convergence speed with gated structure include Reset Gate and Update Gate to store the necessary information from the past, filter out the useless information, and update the current state.^[^ ^35^ ^]^ The raw respiration signals are segmented into frames by applying a sliding window of 30 s. From each frame, the relative capacitance gain dC is extracted as the set of input features to the ML algorithm. Given the sampling frequency 20 Hz of the device, the input of the model dC is with the length of 600 sample points. Each sample point is sequentially processed by the 5 layers of GRUs to obtain the hidden states in RC in the final layer. The final hidden state is concatenated with the max‐pooled feature over all other hidden states in the final layer to acquire the global feature, which is finally passed into a single layer of MLP followed by a softmax layer to predict the probability of each category. With the feature set extracted from the training samples and the corresponding annotated labels, our GRU based machine learning classifier was trained. This trained classifier was applied to the test set for prediction and validation. An example of the raw respiration signals in testing is provided in Figure 4d, and the associated classification results are shown in Figure 4e. Each moving frame window covers a time duration of 30 s with an overlapping of 50%. The domain physiological status can be revealed in the upper part of the graph as we have defined an event as “True” if the relative probability exceeds 50%. For instance, during the sitting and sleeping conditions, the probability of being stays quite beyond 50% and drops during the speaking and exercising conditions. The coughing events (marked with dash lines) are recorded along with following higher coughing probabilities. This function enables classification of body‐active levels from quite to active status and cough alert notifications. Note that the cough duration is less than the output duration as a coughing event can affect the two frames before and after. The results indicate an average sensitivity of 90.0% and an average specificity of 92.5%. The overall accuracy is 92.0%, indicating the potential abilities to be used for the individual daily and medical requirements. Note that the quiet and active events are classified as a non‐coughing status compared with the coughing activity, and the accuracy of the algorithm or medical validation can be further improved with a lower number of confounding activities to train the algorithm (e.g., sneezing and snoring). More details are described in Note S4, Supporting Information.

### 2.5 Applications in Daily and Medical Detection

**Figure 5:**
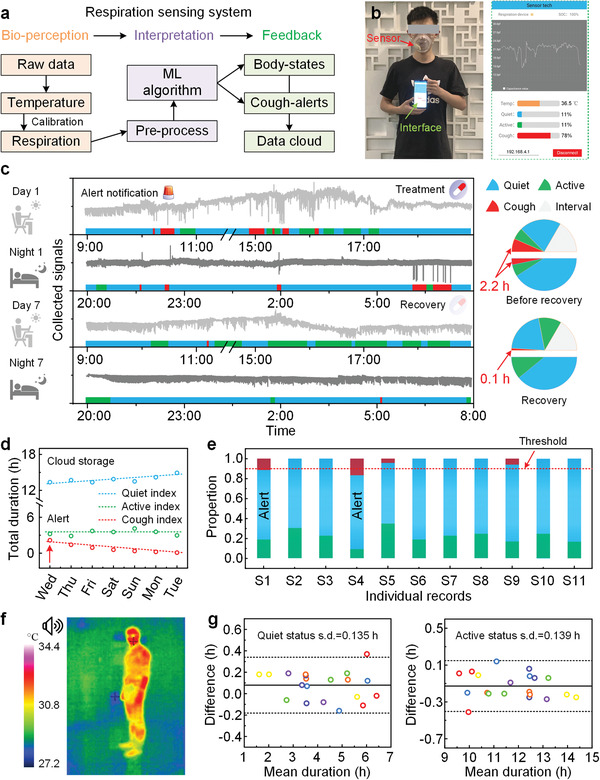
Practical applications of our designed sensing system. a) Operational workflow of the system. b) Optical image of the volunteer wearing the device with displayed user interface. c) Medical monitoring of the cough patient from receiving device alert notification and medical treatment to recovery. d) The time duration data of patient body status in one week, which is stored on cloud clients. e) Group recording data everyday using the device for mass detection. These devices are connected to a shared server and provide each user with their own information. f) Collected data from infrared imaging methods. An audio equipment is also used for accurate reference value. g) The Bland–Altman analysis for quiet and active status with comparisons to the imaging reference measurements. The solid and dashed lines represent mean difference and 1.96 × s.d. of the difference between device measurements and reference values, respectively. Different colors represent the 20 subjects.

To demonstrate the versatility of our proposed ML model and its enabling application, we illustrate its utility from hardware design to software implementation (**Figure** **5**a). This system continuously perceives the respiration signals as raw data, and temperature as data calibration. The raw data would be pre‐processed, including transformation and relative capacitance gain, before entering the interpretation system. Then the ML algorithm mode receives the time domain data to classify the represented body status, as well as the possible coughing alerts. These user data can be stored in cloud client for long‐time monitoring needs and big data analysis.^[^ ^36^ ^]^ Figure 5b shows the matching use of our device and a mobile phone. The user interface displays not only the interpreted physiological parameters but also the real‐time respiration curve with adjustable detecting range. More details are presented in Video S1, Supporting Information, for the software introductions. Note that, the refresh frequency of output index can be depended on the computing abilities of the mobile clients and we set it to be 5 s (83% overlapping of the neighboring frames) to match the temperature setting. The device can provide long time continuous monitoring with high quality repeatability (Figure S11, Supporting Information) and broadcast alert notifications on the user interface as demonstrated in Figure 5c. A user received medication from day 1 is used as an example. The therapeutic effect of the drug can be revealed by the total coughing duration and provides reference for adjustment of dosage. On day 7, the total coughing duration has decreased to be 0.1 h, indicating an effective treatment on this patient with quantitative standards. The dominating body status and total duration statistics on day 1 and day 7 are also presented as the inset color pillar and pie charts. To review the entire recovery process, records are plotted in Figure 5d, where stable and effective treatment effect can be reflected by the gradually decreasing total coughing time counted. Treatment or dosage needs to be changed when the red curve shows the opposite trend. The sensing system can simultaneously monitor many users (Figure 5e), as more than one mobile device can be wirelessly connected to the device at the same time with optional choice to uploading these data to cloud storages. The computing can also be conducted on the cloud instead of the mobiles to reduce the computing burden. Under this scenario, all the devices can be connected directly to the server network without data transferring through mobile clients. Each statistical result will be tagged (S1 to S11 represent 11 anonymous subjects), analyzed and stored in units of days. The subject would receive an alert notification if the coughing level is beyond a certain threshold (0.1 h in this demonstrated frame). We have envisioned more helpful notifications can be provided to users, for instance, a reminder to exercise or fatigue warning. To verify the potential performance, a comparison between a tradition manual labelled method and respiration sensing method is conducted. We use an infrared thermal imager and a synchronized audio equipment to identify their body status (Figure 5f). The monitoring duration totally lasts for 18 h for each participant, mainly consists of a quiet and active body status except for coughing. The body status can be easily distinguished by the visual and sound classification. Similar approaches are often used to examine patients’ postoperative recovery conditions.^[^ ^37^ ^]^ However, such immovable equipment can only be used for fix‐point monitoring and the results rely on manual judgement and statistics. Figure 5g shows Bland–Altman plots for an active and quiet duration time collected by respiration device measurements with comparisons to the imaging reference. The active time duration has a mean difference of 0.079 h and a s.d. (standard deviation) of the difference between respiration measurement and imaging reference of 0.135 h. The quiet time duration has a mean difference of −0.130 h and a s.d. of 0.139 h. The solid and dashed lines represent mean difference and 1.96 × s.d. (95% limits of agreement) of the difference between device measurements and reference measurements, respectively. To emphasize, this limit of agreement is comparable to that observed with manual labelled results for body status monitoring. The slight difference might be attributed to the vague classification rules of visual monitoring. Benefitted by the respiration sensing system, body status can be efficiently classified and counted without limiting the users.

## 3 Conclusion

Here, we have developed a soft, wireless, and non‐invasive sensing system for quantitative and real‐time evaluation of respiration signals. The system consists of hardware design, data analytics, and software‐based algorithmic model deployment. Customized capacitive and resistive sensors are applied to simultaneously capture respiration and temperature signatures with high data fidelity. To seek the relationship between physiological status and respiration, we further propose a machine learning‐based respiration framework with a set of carefully studied features as inputs, and a high recognition accuracy of over 90% is achieved. We embedded the model into mobile clients connected to the device network, and the body status can be classified accurately using our model and reflected on the user interfaces. The device can provide users with quantitative data on the duration of physiological activities, health status advice, and alert notifications. Moreover, the sensing system has multiple access points and each point can be adapted to at least one user to monitor the respiratory health. The collected data can be quantitatively analyzed and stored to effectively indicate the effect of epidemic prevention policy and treatment on respiratory‐related diseases. Overall, this work offers broad capabilities relevant to applications ranging from medical assistance to daily physiological detection.

## 4 Experimental Section

### Fabrication of Sensors

The initial polyimide film (13 µm thick, on polyethylene terephthalate supporting layer) was fixed onto the chamber substrate holder with an attached mask (stainless steel with thickness of 0.03 mm) under an electron beam evaporation workstation (TF500‐HHV). The hollowed‐out serpentine lines were patterned into geometries on the metal mask by a CO_2_ laser cutter (VLS3.50). During the sputtering process, the flow rate of argon (Ar, 99.999%) and the working pressure were fixed at 20 sccm and 0.440 Pa. Copper films with regulated thickness were sputtered onto the PET film. An extra thin Au layer (thickness of 1–2 nm) was followed to provide oxidation resistance and better weldability. The patterned electrodes then appeared after removing the attached masks. The fibers were fabricated using a TL‐Pro electrospinning machine (Tong Li Weina Technology Co., Ltd., Shenzhen, Guangdong, China) using a 20‐gauge blunt steel needle. Thermoplastic polyurethanes (TPU, 9380AU) particles were dissolved in N,N‐dimethylformamide (7/3 v/v) with vigorous magnetic stirring to obtain a clear solution with a concentration of 10% (w/v). Spinning was carried out at a flow rate of 1 mL h^−1^, applied voltage of 20 kV, and a collecting distance of 20 cm. The fabricated TPU layer was hydrophobic with contact angle of 103° (Figure S12, Supporting Information), which effectively protected the sensor and reduced the sensing bias shift after wearing them for a period of time.

### Fabrication of Electronics

The LDO was fabricated in SMIC 180 nm CMOS process, which occupies a chip area of 0.046 mm^2^, with the die microphotograph. In this study, the LDO can support an input voltage range of 0.8–3.3 V and an output voltage range of 0.6–3.2 V. The maximum load current of the LDO was 201 mA with an external capacitance of 1 µF for testing purposes and no internal capacitor was implemented on‐chip. The average parameters of amplifier DC gain, unity‐gain bandwidth, phase margin and slew rate were 94.18 dB, 4.81 MHz, 58.42° and 2.53 V µs^−1^, respectively. Besides, the error amplifier consumes little quiescent current and the maximum current efficiency of the LDO was 99.99%. Other commercial chips were also assembled on a sheet of fPCB into compact layouts. Solder paste (Chip Quik TS391LT) and a heat gun (AOYUE Int866) joined the various electronics and sensors onto the fPCB.

### Silicone Protections and Assembling

A three‐axis milling machine created aluminum molds in geometries, defined by 3D computer‐aided design drawings created on ProE Creo 3.0. The elastomeric protection was formed by casting a silicone thermoset polymer into the gap formed by matching pairs of molds (Ecoflex, 00–30, smooth‐on) with thin thickness (≈0.3 mm). Curing occurred in an oven at 70 °C for 15 min. The cavity that enclosed the electronics was defined by bonding this protection to a planar silicone substrate film around the perimeter. This design provided a waterproof encapsulation structure that also allowed for the free movement of the buckled serpentine lines. A layer of a soft silicone gel (Ecoflex, smooth‐on) served as a degree of mechanical isolation from the underlying object. The gel cured at room temperature in less than 30 min. The assembling and bonding were realized after silicone curing and assisted by an adhesive (loctite tak pak 444). The device showed no degradation in performance after complete immersion in phosphate buffered saline solution at 70 °C for ten days. Relative cost details are presented in Figure S13, Supporting Information.

### Simulation and Characterizations of the Devices

Simulation of the touching and pressing process was carried out using solid mechanics and electrical current module in COMSOL Multiphysics 5.4. Both sensors were set on a flexible substrate with one end constraint. The torque force was simulated by a couple of rotationally symmetric force. The micro structure of encapsulating layer was observed by a field emission scanning electron microscope (FESEM) (GeminiSEM 500, ZEISS). The resistance and capacitance were measured by a TongHui H2832 Precision LCR meter.

### Data Analytics and Software Design

The participants were all recruited by the School of Electrical and Electronic Engineering Nanyang Technological University and entirely voluntarily. For all the studies, the participant gave informed consent. The devices used were considered to carry minimal risk, and therefore approval was not needed. Collected data analysis was performed on MATLAB (R2018b) with technical computing language. The machine learning model was built by the PyTorch 1.10.1 machine learning library with Python 3.7. The model was trained by 200 epochs using the AdamW optimizer and the cosine learning rate scheduler with an initial learning rate 1.0 × 10^−5^. The ML model was converted into a lightweight version and deployed into Android on Android Studio (2021.1.1.23) with PyTorch Android lite library. The displayed mobile clients included a Samsung A52s (equipped with Android 12 system) and a Redmi 9A (equipped with Android 10 system).

### Statistical Analysis

The initial quantitative analysis results of sensing parameters were obtained from 5 samples. The respiration classification dataset containing respiration data was collected from 11 subjects to train and validate the proposed ML algorithm. The relative capacitance gain was applied as data processing methods. The confirmatory study validates the device performance and the algorithm in a cohort of volunteers (*n* = 13). Bland‐Altman plots for active and quiet duration time were obtained by respiration device measurements with comparisons to the imaging reference. The active time duration had a mean difference of 0.079 h and a s.d. (standard deviation) of the difference between respiration measurement and imaging reference of 0.135 h. The quiet time duration had a mean difference of −0.130 h and a s.d. of 0.139 h. The statistical analysis was assisted by Origin (2019b).

## Conflict of Interest

The authors declare no conflict of interest.

## Supporting information

Supporting Information

Supplemental Video 1

Supplemental Video 2

## Data Availability

The data that support the findings of this study are available in the supplementary material of this article.
