# Supplementary material for: Quantitative and Real‐Time Evaluation of Human Respiration Signals with a Shape‐Conformal Wireless Sensing System
Source: Adv Sci (Weinh). 2022 Sep 11;9(32):2203460. doi: 10.1002/advs.202203460 (PMC9661834; doi:10.1002/advs.202203460)
Supplement: Supplementary file 1 — Supporting Information [file ADVS-9-2203460-s003.pdf]

Quantitative and real-time evaluation of human respiration signals using a shape-conformal wireless sensing system

Sicheng Chen[1,&,*], Guocheng Qian[2,&], Bernard Ghanem[2], Yongqing Wang[3], Zhou Shu[1], Xuefeng Zhao[4], Lei Yang[5], Xinqin Liao[6] and Yuanjin Zheng[1]*

[1]School of Electrical and Electronic Engineering Nanyang Technological University, 50 Nanyang Avenue, Singapore 639798, Singapore

[2]Visual Computing Center, King Abdullah University of Science and Technology, Thuwal 23955-6900. Kingdom of Saudi Arabia

[3]School of Geophysics and Information Technology, China University of Geosciences, Beijing 100084, China

[4]Shanghai Institute of Intelligent Electronics & Systems, School of Microelectronics, Fudan University, Shanghai 200433, China

[5]Key Laboratory of Education Ministry for Modern Design and Rotor-Bearing System, Xi’an Jiaotong University, Xi'an 710049, China

[6]Shanghai Institute of Intelligent Electronics & Systems, School of Microelectronics, Fudan University, Shanghai 200433, China

[&]These authors contributed equally

*Corresponding authors, sicheng.chen@ntu.edu.sg; yjzheng@ntu.edu.sg

Supplementary Information

Note S1. System Operation Flow..3

Note S2. The theoretical working model of capacitive sensor..3

Note S3. Temperature calibration of the respiration sensor...4

Note S4. The model validation parameters..4

Fig. S1. Stable electrical interconnects under the strain of 10%..5

Fig. S2. Step-by-step electron beam evaporation technology..6

Fig. S3. Sensing structures with serpentine-shaped patterns...7

Fig. S4. Sensing structures with less-serpentine design...8

Fig. S5. Output sensor data with less-serpentine design...9

Fig. S6. The general layouts of fPCB..10

Fig. S7. Sensitive peripheral resistance-reading circuits..11

Fig. S8. Sensitive peripheral capacitive-reading circuits..12

Fig. S9. Signals collected from diverse subjects and devices...13

Fig. S10. The data outputs before and after sensor deformation...14

Fig. S11. The excellent repeatability in our long-time monitoring on practical applications......15

Fig. S12. The contact angle of encapsulation TPU layer...16

Fig. S13. The cost details of our sensing system..17

Note S1. System Operation Flow

Device operation: The user interface controls the device operation. To turn on the system, it first connects to the IP address of the device. By default, the address is 192.168.4.1 (defined by the AP setting) and the device starts working after pressing the CONNECT button. A second pressing-on action would disconnect the device. The user interface starts and stops recording the data to an onboard flash memory within the device or uploading the data to cloud client if needed. When the session begins, the MCU receives the data from the reading circuit and writes it to the flash memory. During the recording session, users can monitor the multi-index and real-time curve anywhere as long as an equipment is connected to the AP internet.

Power-flow: The power from Li-ion battery goes through the LDO (DC/DC converter) that regulates it to 3.3 V and delivers to the active components throughout the system, which includes a microcontroller (MCU), paired sensors, reading IC (Fig. S5 and general fPCB layouts in Fig. S6) and wireless communication circuits.

Note S2. The theoretical working model of capacitive sensor

Due to the parallel relationship between initial capacitance and additive resistance, the total impendence *Z* under passing gas molecules can be expressed as

$$\frac{1}{Z}=\frac{1}{R}+j\omega C_0$$

So we can find

$$Z=\frac{R}{1+R^2\omega^2{C_0}^2}-j\frac{R^2\omega C_0}{1+R^2\omega^2{C_0}^2}=R'+\frac{1}{j\omega C'}$$

We can thus obtain an imaginary part (capacitive value) as

$$\frac{1}{j\omega C'}=-j\frac{R^2\omega C_0}{1+R^2\omega^2{C_0}^2}$$

The above equation can be simplified to

$$\frac{1}{\omega C'}=\frac{R^2\omega C_0}{1+R^2\omega^2{C_0}^2}$$

Thus obtain an updated capacitive value as

$$C'=C_0+\frac{1}{R^2\omega^2C_0}$$

Finally, the normalized capacitance change for the device can be calculated as

$$\frac{\Delta C}{C_0}=\frac{1}{R^2\omega^2{C_0}^2}$$

We can see that relative the capacitance fluctuates along with the parallel resistance, induced by the human respiration process.

Note S3. Temperature calibration of the respiration sensor

For the 100-nm thick respiration sensor, as shown in Fig.2g, the capacitance errors are linear between 25-50 °C (T). The error is calculated to be c.a. 0.01% in this range. If we define the initial capacitance value as C_0, the changed capacitance C should be

$$C = C_0 \times (1 + 0.01\% \times (T - 25))$$

That is

$$C = C_0 \times (0.01\%T + 99.75\%)$$

While T can be calculated according to the resistance output, thus the corrected output C_x should be

$$C_x = C/(0.01\%T + 99.75\%)$$

In most occasions, such correction is not necessary especially for the physiological temperature range. Nevertheless, all the temperature data would be saved to the memory for the assisted respiration analysis. This method would be useful in other potential applications under extreme temperature conditions.

Note S4. The model validation parameters

The sensitivity (True Positive Rate) refers to the probability of a positive test, conditioned on truly being positive. Thus 9 true subjects among 10 detected cough patients means a 9/10 sensitivity. The Specificity (True Negative Rate) refers to the probability of a negative test, conditioned on truly being negative. Thus 37 true subjects among 40 detected non-cough patients means a 37/40 sensitivity.

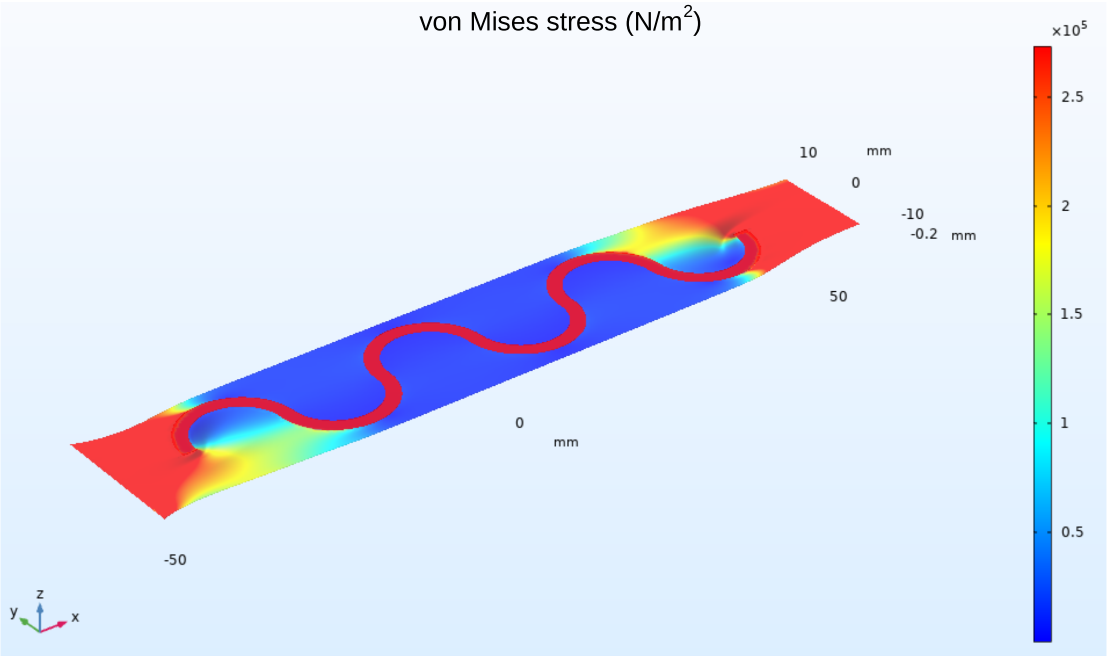

Fig. S1. Stable electrical interconnects under the strain of 10% (10-mm stretching length of a 100-mm long interconnect with serpentine pattern). The yield strength of Copper (Cu) is c.a. 350 Mpa, which is much larger than the maximum value in above simulation results.

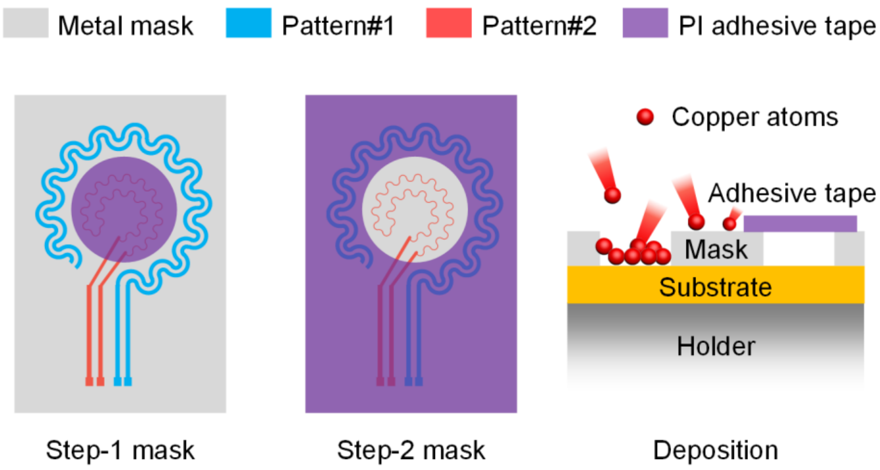

Fig. S2. Step-by-step electron beam evaporation technology (The metal mask was partially covered by PI adhesive tape in step-1 and step-2, respectively, followed by the layer-deposition process. Note that, such method aims to build a thickness difference between different sensors on same substrate with acceptable positioning accuracy. The thickness should meet certain requirement from giving sensor type.eg, capacitive sensor with 1 μm thickness and resistive sensor with 0.1 μm thickness)

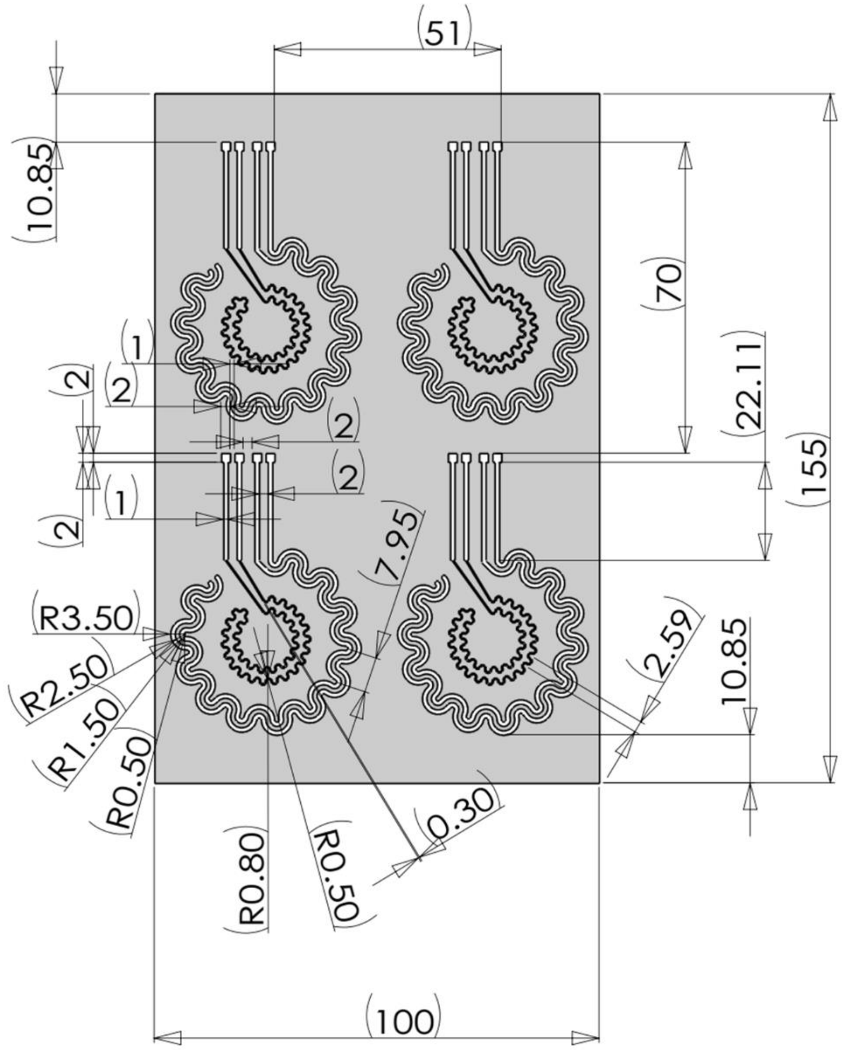

Fig. S3. Sensing structures with serpentine-shaped patterns (Both sensors are concentric to support suitable temperature correction functions. The mask is stainless metal with thickness of 30 μm, graved by a laser cutter)

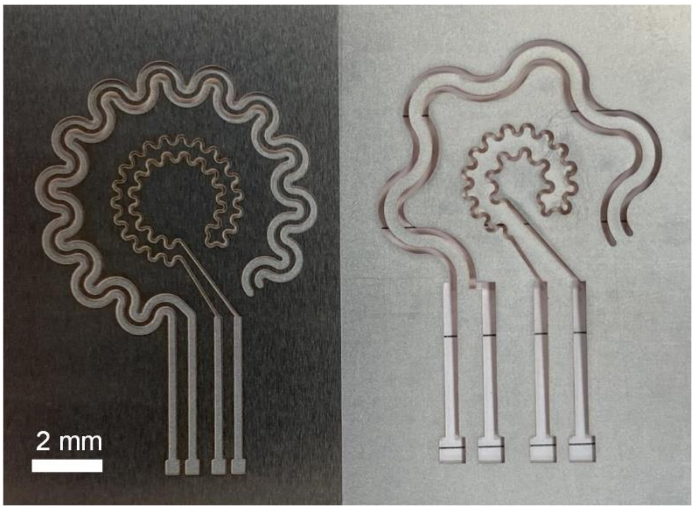

Fig. S4. Sensing structures with less-serpentine design (The circular distribution of these serpentine structures allows more sensitive collection of respiration signals within certain area. Moreover, the parallel resistance can be obviously regulated with more serpentine lines. More details can be found in Note S2 and our recent work [25]).

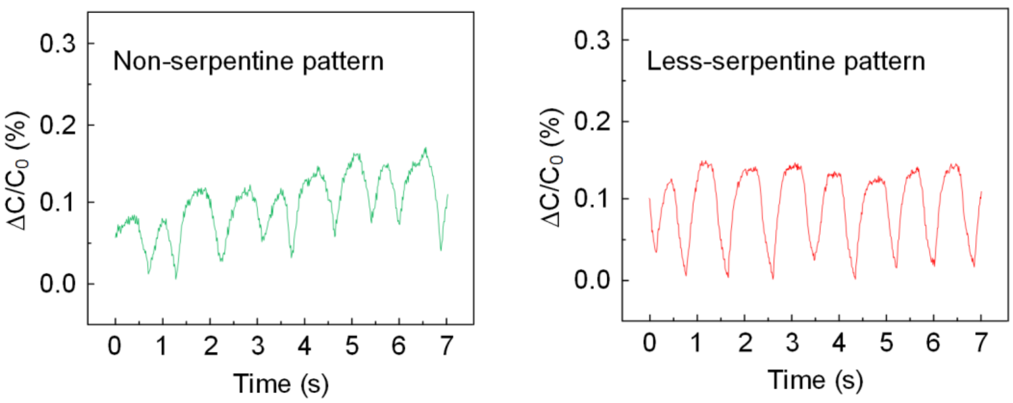

Fig. S5. Output sensor data with less-serpentine design (The Non-serpentine pattern and less- serpentine pattern show less sensitive response to the respiration signals and possess higher noise background in the peak position compared the pattern applied in the device).

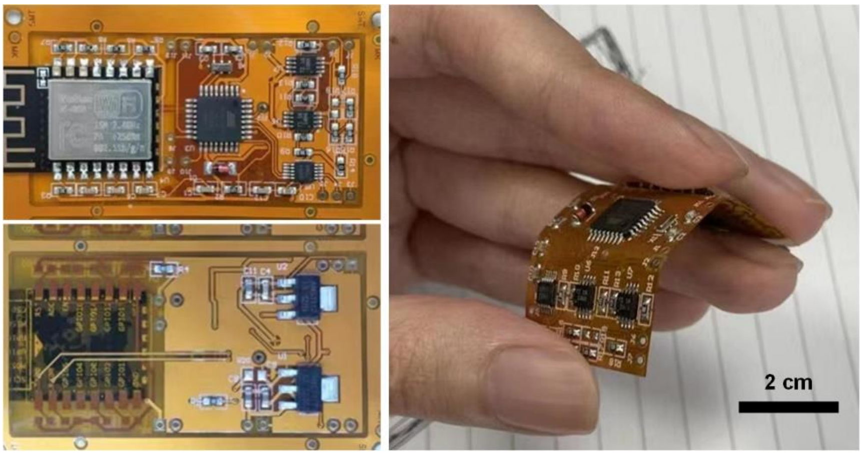

Fig. S6. The general layouts of fPCB. (This is a protype of our designed board integrated with customized and commercial chips. The board can bear bending and twisting deformations with high stability and reliability.)

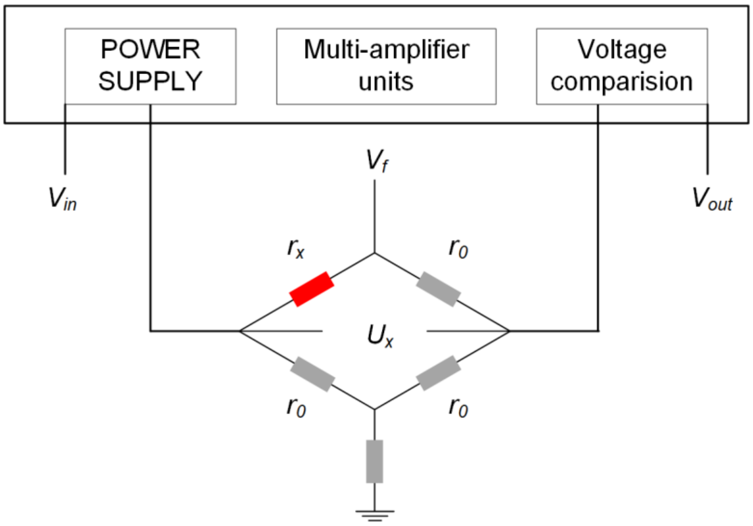

Fig. S7. Sensitive peripheral resistance-reading circuits (multi-stage amplifier is used combing with bridge structure, where the matching resistor value decides the balance point of the bridge and should be chosen properly. As for the balance conditions, the U_x equals to zero and it would make a huge difference once the r_x changes).

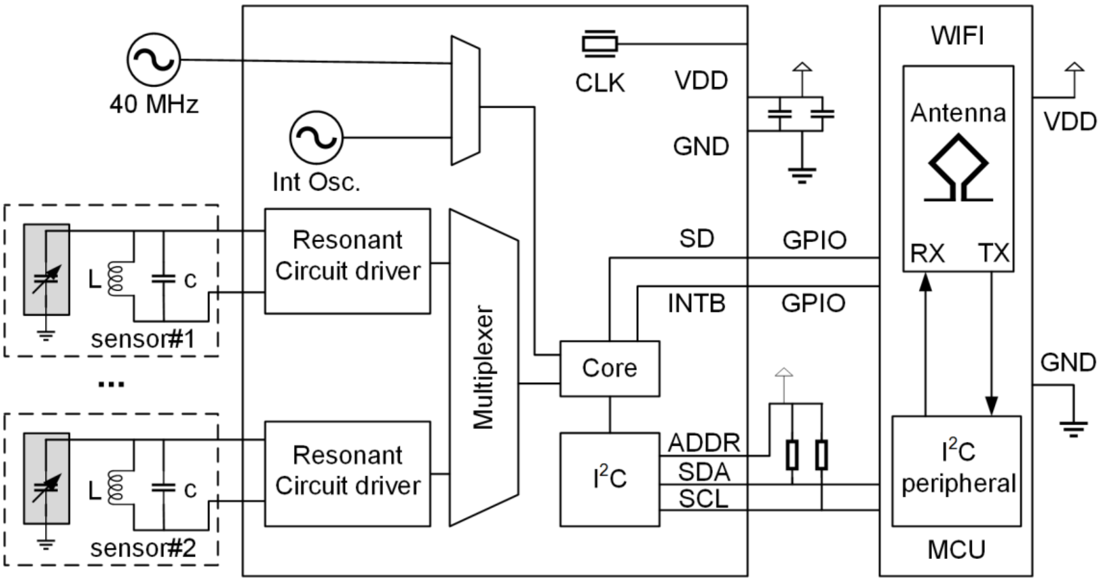

Fig. S8. Sensitive peripheral capacitive-reading circuits. (The single-end connections are applied with shared ground and the frequency shift can reveal the capacitance fluctuations as delivered to the MCU unit for reading.)

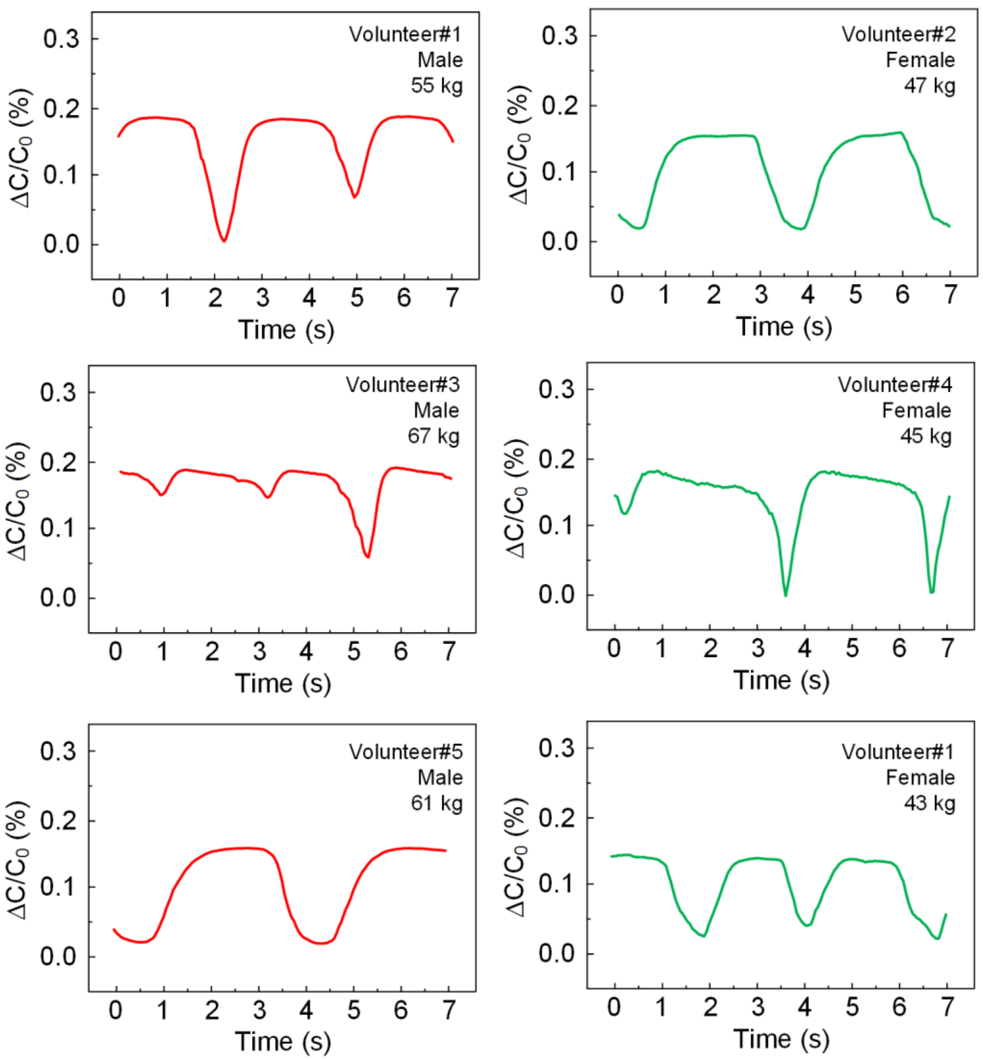

Fig. S9. Signals collected from diverse subjects and devices. (to uniform the testing preconditions, these signals are all collected under their rest. These signals are all chosen randomly and we can see that the amplitude and baseline are diverse, thus hindering the model training process.)

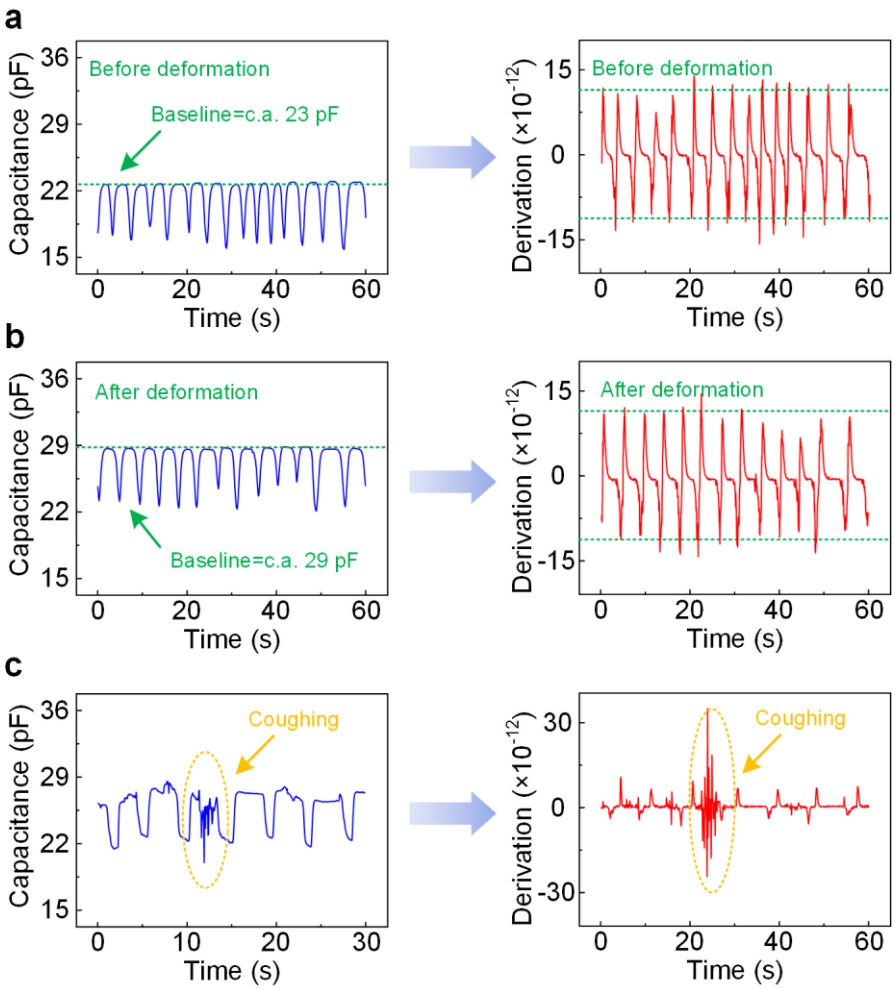

Fig. S10. The data outputs before and after sensor deformation with corresponding data processing. (a-b: the maximum deformation is under 30° bending angle. We can see that, although baseline-shift occurs on the sensing curve of raw data from c.a. 23 pF to c.a. 29 pF induced by large sensor deformations, the following first step of data processing would effectively correct this factor to ensure the correct classification of respiration signals using our ML algorithm. That is to say, the respiration sensor would be more sensitive to the relative changes rather than shape deformation. c: as for the coughing detection, we take the sensor with bending deformation in a mask as an example, the processed data would also be classified into coughing status. This result can also be found in our supplementary video S2 (the rest status mixed with coughing), where the sensors are bent to fit the shape of mask.)

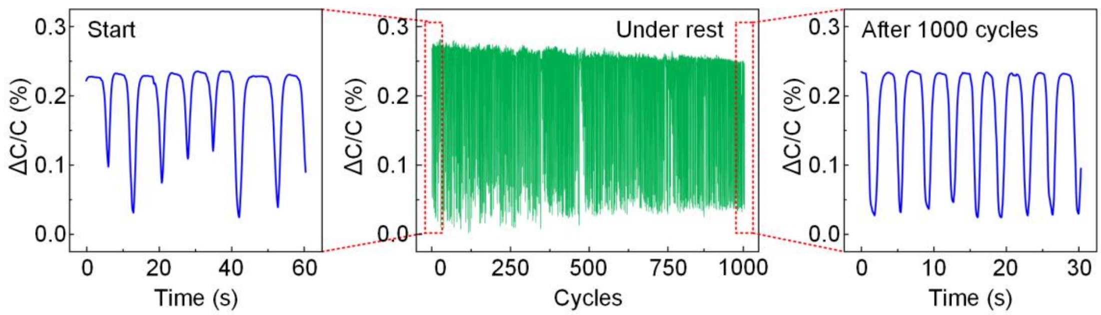

Fig. S11. The excellent repeatability in our long-time monitoring on practical applications. (we use the sensor to collect over 1000 breaths and see that the sensor can still remain stable performance under respirations.)

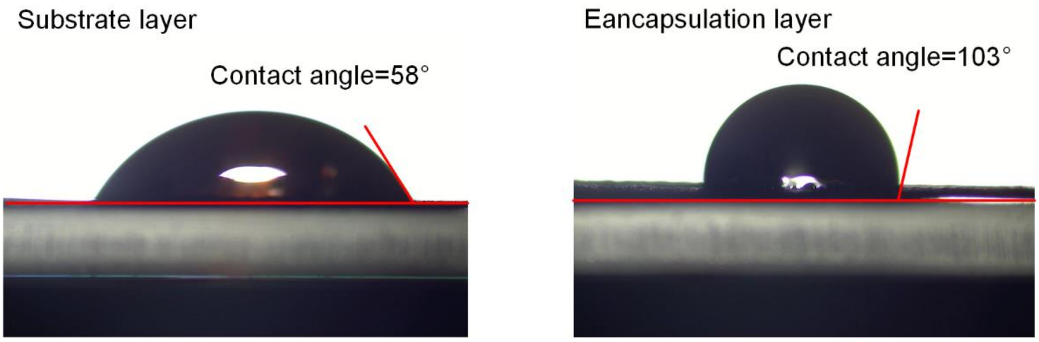

Fig. S12. The contact angle of encapsulation TPU layer. (We applied two methods to reduce the abnormal humidity influence. One is to assemble a breathable TPU layer upon sensor substrates (The substrate is more easily affected by humidity due to its hydrophilic properties after oxygen treatment during deposition). The hydrophobic TPU would effectively protect the sensor and reduce the sensing bias shift after wearing them for a period of time. The other one is improving by our ML algorithmic model and data processing. The derivation process would focus more on the respiration trend and relative value gain despite of some inevitable bias shift caused by abnormal humidity conditions.)

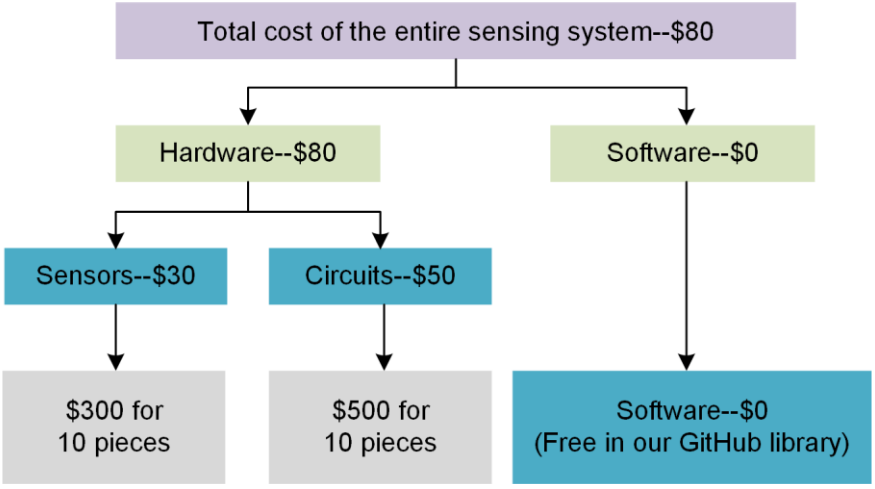

Fig. S13. The cost details of our sensing system. (Although the sensors have good stabilities benefitted by its non-invasive design, we do not recommend continuous use for more than a week which might may cause bacterial growth. Of course, cross-use between different users is also not recommended.)
